# Identification of Interleukin-8-Reducing Lead Compounds Based on SAR Studies on Dihydrochalcone-Related Compounds in Human Gingival Fibroblasts (HGF-1 cells) In Vitro

**DOI:** 10.3390/molecules25061382

**Published:** 2020-03-18

**Authors:** Katharina Schueller, Joachim Hans, Stefanie Pfeiffer, Jessica Walker, Jakob P. Ley, Veronika Somoza

**Affiliations:** 1 Department of Physiological Chemistry, Faculty of Chemistry, University of Vienna, Althanstraße 14, 1090 Vienna, Austria; Katharina.zakovsek@gmx.at (K.S.); Stefanie.pfeiffer88@gmx.at (S.P.); veronika.somoza@univie.ac.at (V.S.); 2 Symrise AG, Mühlenfeldstraße, 37603 Holzminden, Germany; joachim.hans@symrise.com (J.H.); jakob.ley@symrise.com (J.P.L.); 3 Department of Analytical Chemistry, Faculty of Chemistry, University of Vienna, Währinger Straße 38, 1090 Vienna, Austria; 4 Leibniz Institute for Food Systems Biology at the Technical University of Munich, Lise-Meitner-Straße 34, 85354 Freising, Germany

**Keywords:** structure–activity relationship, inflammation, dihydrochalcones, IL-8, HGF-1 cells

## Abstract

**Background:** In order to identify potential activities against periodontal diseases, eighteen dihydrochalcones and structurally related compounds were tested in an established biological in vitro cell model of periodontal inflammation using human gingival fibroblasts (HGF-1 cells). Methods: Subsequently to co-incubation of HGF-1 cells with a bacterial endotoxin (*Porphyromonas gingivalis* lipopolysaccharide, *pg*LPS) and each individual dihydrochalcone in a concentration range of 1 µM to 100 µM, gene expression of interleukin-8 (IL-8) was determined by qPCR and cellular interleukin-8 (IL-8) release by ELISA. **Results**: Structure–activity analysis based on the dihydrochalcone backbone and various substitution patterns at its aromatic ring revealed moieties 2′,4,4′,6′-tetrahydroxy 3-methoxydihydrochalcone (**7**) to be the most effective anti-inflammatory compound, reducing the *pg*LPS-induced IL-8 release concentration between 1 µM and 100 µM up to 94%. In general, a 2,4,6-trihydroxy substitution at the A-ring and concomitant vanilloyl (4-hydroxy-3-methoxy) pattern at the B-ring revealed to be preferable for IL-8 release inhibition. Furthermore, the introduction of an electronegative atom in the A,B-linker chain led to an increased anti-inflammatory activity, shown by the potency of 4-hydroxybenzoic acid *N*-vanillylamide (**13**). **Conclusions:** Our data may be feasible to be used for further lead structure designs for the development of potent anti-inflammatory additives in oral care products.

## 1. Introduction

Periodontal diseases are a significant health problem with a high prevalence and incidence worldwide, and have a major impact on the quality of life [1]. Gingivitis is the first stage in the development of periodontitis and starts at the free gingival margin, with a reversible inflammatory immune reaction to accumulated dental plaque caused by a lack of oral hygiene [2]. Clinical signs of gingivitis are changes of tissue color and texture, bleeding upon gentle probing and over-production of crevicular fluid [2]. If untreated, the persisting immune reaction to plaque bacteria can promote loss of the attaching tissue and the formation of periodontal pockets [2]. In the worst case, periodontitis can peak in the infestation of the alveolar bone and tooth loss [2]. In the Netherlands, a western country with high hygienic standards, dentists surveyed that 32% of their patients suffered from gingivitis [3]. One strategy to counteract periodontal inflammation is the addition of anti-inflammatory compounds to oral care products. For example, the authors previously showed an anti-inflammatory impact on oral inflammation by the addition of a plant extract to chewing gum [4].

Especially, natural compounds have been used in various food and cosmetic products due to their beneficial health effects. Neohesperidin dihydrochalcone (NHDC, **1**), a high impact sweetener approved for the application in foods, beverages, and oral hygiene products, is structurally related to polyphenols, which are widely known for their anti-inflammatory activity [5,6,7]. NHDC, some related dihydrochalcones, and the glycosylated compound hesperidin (**3**) have been tested for their anti-inflammatory potential [8,9,10,11]. The dihydrochalcone phloretin, naturally found in apples, is known to inhibit the mRNA expression of pro-inflammatory genes, such as nuclear factor kappa B (Nf-κB) or interleukin-8 (IL-8), in human cell lines [12] and reduced the lipopolysaccharide-induced inflammatory response in a murine cell model of systemic inflammation [13]. However, there are neither data on periodontal disease models nor in-depth studies on structural requirements for superior anti-inflammatory activity of dihydrochalcones and related compounds.

The screening of an anti-inflammatory potential of dihydrochalcones with regards to oral inflammation requires an appropriate model system. One of the bacteria strongly associated with periodontal diseases is the gram-negative germ *Porphyromonas gingivalis* (*pg*) [14]. Bacteria and their side-products (endotoxins and membrane components) are recognized by cells *via* pattern recognition receptors, with toll-like receptors (TLR) among those [14]. Lipopolysaccharides (LPS) are a type of bacterial endotoxins from the outer bacterial membrane and trigger TLR-mediated activation of transcription factor Nf-κB in gum tissue, resulting in the release of inflammatory cytokines and chemokines [14]. IL-8, belonging to the family of CXC motif chemokines, is essential in the response to infection and injury by activating various cell types surrounding the inflamed site and recruiting immune cells from the blood stream [15]. The in vitro release of IL-8 in human gingival fibroblasts (HGF-1), a well-established model of gum inflammation, can be up-regulated to up to 40-fold by stimuli like *Porphyromonas gingivalis* lipopolysaccharide (*pg*LPS) [6]. The addition of anti-inflammatory active compounds in this model decreased the LPS-stimulated release of pro-inflammatory markers, like IL-6, IL-8, and monocyte chemotactic protein 1 (MCP-1) [6,16,17]. In order to continuously impair gum inflammation, caused by plaque bacteria, the supplementation of oral hygiene products with antimicrobial and anti-inflammatory natural and synthetic substances is a common strategy in the prevention and treatment of gum diseases [18,19,20]. In addition, for hesperitin, an aglycone structurally related to neohesperdin, the mode of its anti-inflammatory activity has been investigated intensively, demonstrating both, a modulation of the TLR4/Nf-κB signaling pathway [21] as well as an impact on the mitogen-activated protein kinase pathway [22]. Both pathways are known to be involved in the inflammatory response to lipopolysaccharides [23,24] and are targeted by anti-inflammatory polyphenols [25,26]. The red-wine polyphenol resveratrol possesses its anti-inflammatory activity by inhibiting these pathways, potentially by directly interacting with TLR4 [27], suggesting a potential mechanism of action for anti-inflammatory test compounds.

The aim of the present study was the identification of the anti-inflammatory effects of polyphenolic compounds regarding oral inflammation. Based on the dihydrochalcone structure of **1**, a known anti-inflammatory compound, we developed a substance library consisting of 18 compounds (Table 1) and subjected them to testing in an in vitro model of *pg*LPS-induced inflammation in human gingival fibroblasts (HGF-1) for the reduction of IL-8 release as a key biomarker of the immune response.

## 2. Results

In this report, structure–activity relationships of dihydrochalcones and related substances are presented, and we propose two structural patterns for IL-8 release reduction to be used as lead structures for future studies.

### 2.1. Compound Category Effectiveness

**Figure 1:**
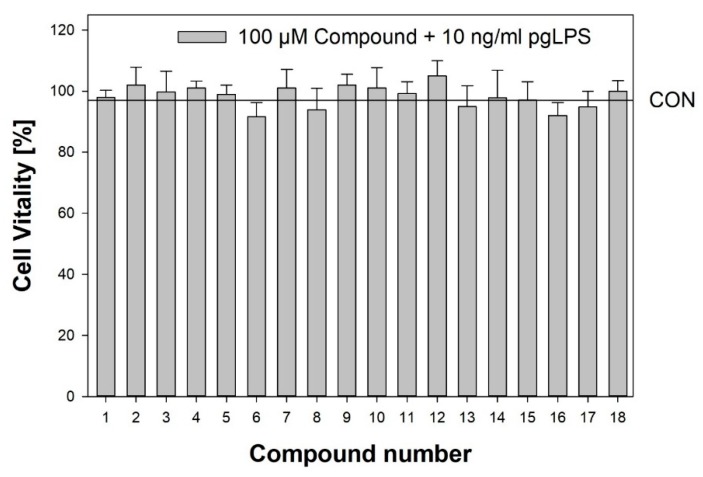
Cell vitality upon 6 h incubation of human gingival fibroblasts (HGF-1) cells with 10 ng/mL lipopolysaccharides (LPS) and 100 µM of the respective compound. Shown are average ±SD compared to untreated cells. The horizontal line marks the vitality of cells treated with 0.1% DMSO only. Significant differences between treatment and control were tested using the Student’s *t*-test.

Substances (Table 1) were tested at five concentrations ranging between 1 µM and 100 µM in an in vitro model previously published [28], with the modifications described in the method section. After 6 h of co-incubation with *pg*LPS and the test compound, IL-8 release was measured in the supernatants, and the calculated percentage change was compared to the inflammatory stimulus *pg*LPS alone. Cell vitality was determined using an MTT assay upon 6 h incubation of the HGF-1 cells with 10 ng/mL *pg*LPS and 1–100 µM of each compound. Treated HGF-1 cells did not show any significant reduction of cell vitality compared to the respective solvent control (0.1% DMSO) as shown in Figure 1 for the compounds **1**–**18** in the highest tested concentration of 100 µM co-incubated with 10 ng/mL *pg*LPS. Therefore, the effects of the tested compounds measured on IL-8 release are not affected by cytotoxic effects. Graphs for the release of IL-8 after incubation with substances **1** to **18** are part of the supporting information (Figure 4, Figures S2 and S3). Subsequently, the area under the curve (AUC) was calculated for each substance (AUC_1,...,18_) and categories of substances (AUC_median_) as the sum of trapezoid areas between data points.

**Figure 2:**
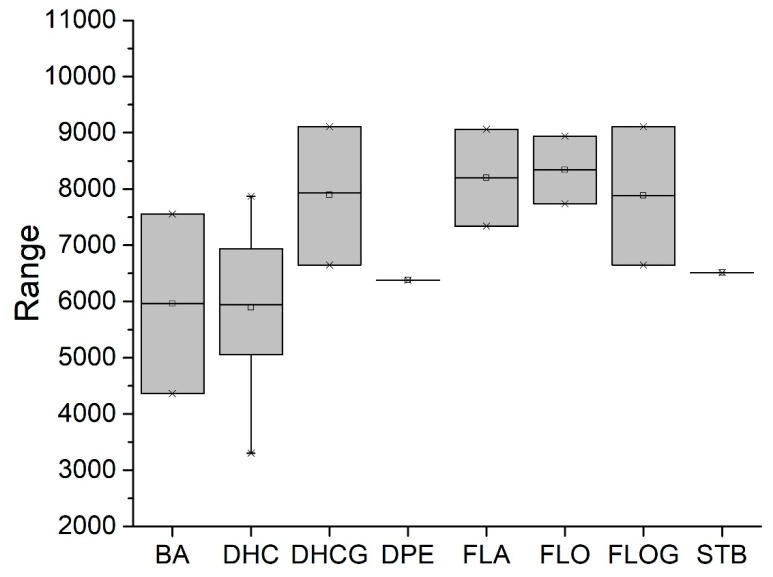
Comparison of the reduction of IL-8 release in *pg*LPS-stimulated HGF-1 cells among groups of the tested compounds (1 to 18) with different core scaffolds. Depicted are boxplots (min, Q_0.25_, median, Q_0.75_, max) of the individual AUCs in the substance groups. Substance groups are N-benzyl-benzamides (BA; *n* = 2; compounds **13** and **14**, AUC_median_ = 5928), dihydrochalcones (DHC; *n* = 7; **6**–**12**, AUC_median_ = 5738), dihydrochalcone glycosides (DHCG; *n* = 3; **1**–**3**, AUC_median_ = 7766), diphenylethanone (DPE; *n* = 1; **17**, AUC = 6378), flavans (FLA; *n* = 2; **15** and **16**, AUC_median_ = 8090), flavanones (FLO; *n* = 2; **4** and **5**, AUC_median_ = 8105), and stilbenoid (ST; *n* = 1; **18**, AUC = 6512).

The difference between single compound values (ΔAUC_x/y_) or between category median values (ΔAUC_median(catx)/median(caty)_) was used for the comparison of effectiveness. Low AUCs therefore mean high bioactivity, and the closer ΔAUC_x/y_ is to zero, the less the substances differ in bioactivity. As our first step in defining SAR, substances were categorized by core structure, resulting in seven structural subclasses (Figure 2), where the class of diphenylethanons (DPE) and stilbenes (STB) only consisted of one compound each (**17** and **18**, respectively).

Considering only groups with at least two compounds, Dihydrochalcones (DHC, *n* = 7, AUC_median_ = 5738) and *N*-benzylamides (BA, *n* = 2, AUC_median_ = 5928) exhibited the most potent effects, as depicted in Figure 2.

### 2.2. Substitution Pattern Analysis of Aromatic Rings A and B

**Figure 3:**
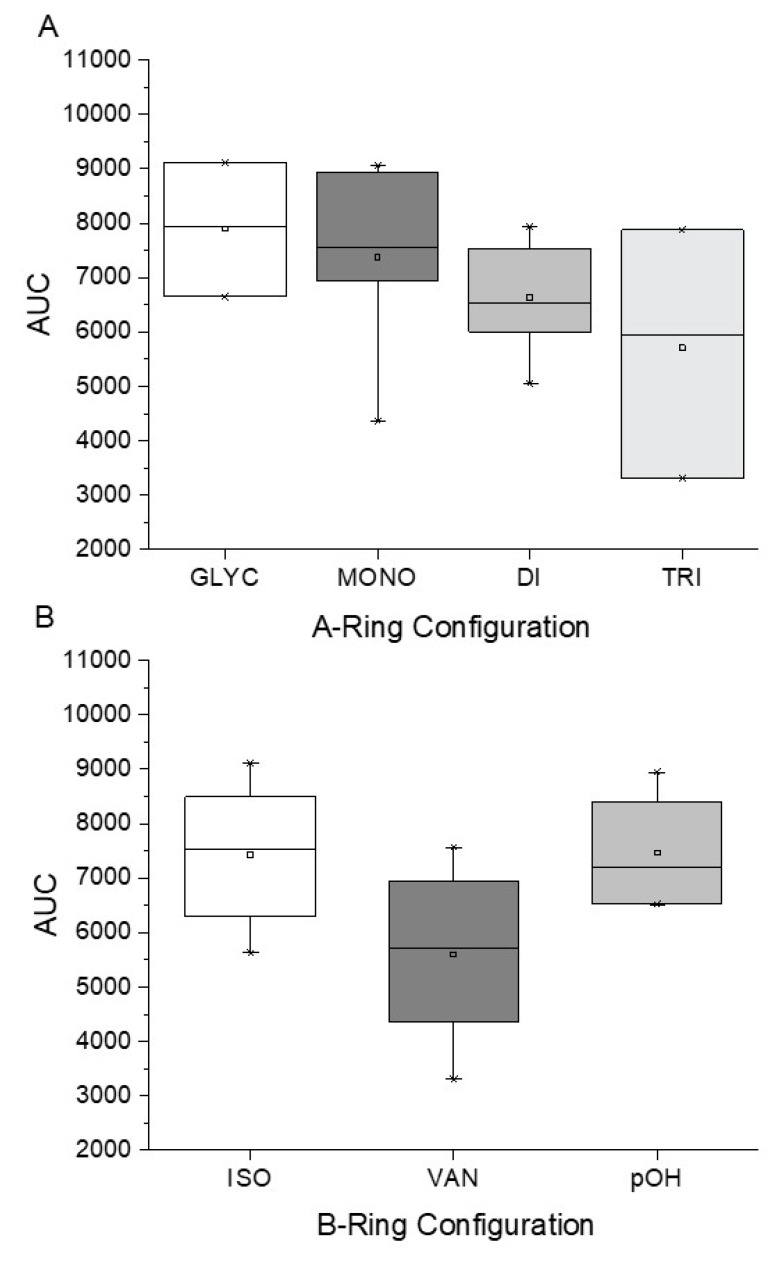
Clustering of compounds **1**–**18** according to structural similarity of (**A**) configuration on the A-ring, with glycoslated (*n* = 3; **1**–**3**), monohydroxylated (*n* = 2; **12** and **13**), dihydroxylated (*n* = 8; **5**, **9**–**11**, **14**, **16**, and **17**), and trihydroxylated (*n* = 8; **1**–**4**, **6**–**8**, **15**) and (**B**) configuration on the B-ring, with isovanilloyl (*n* = 9; **1**–**4**, **6**, **11**, **15**, and **16**), vanilloyl (*n* = 6; **7**, **10**, **12**–**14**, and **17**), and p-hydroxy benzyl (*n* = 4; **5**, **8**, **9**, and **18**) moiety. Depicted are box-plots (min, Q_0.25_, median, Q_0.75_, max) of the respective group AUCs.

To investigate the influence of the number and type of substituents on effectiveness, all data were analyzed for substitution patterns at the aromatic rings A and B (Figure 3A), independent of the base structure having a bicyclic system (**2**–**5**, **15**, **16**) or not (**1**, **6**–**14**, **17**, **18**), or being glycosylated (**1**–**3**) or not (**4**–**18**) (Table 1).

Figure 3A shows that for an IL-8 reducing effect, a trihydroxylated A-ring is preferable (AUC_median_ = 5524), as opposed to any other substitution pattern (AUC_median_ between 6506 and 7769). Concerning the B-ring functional groups, our data suggest the vanilloyl group to be the most active for an anti-inflammatory effect (Figure 3B), with an AUC_median_ = 5464 for the vanilloyl group compared to AUC_median_ = 7290 for the isovanilloyl group and AUC_median_ = 7385 for the p-hydroxy benzyl moiety.

### 2.3. Single Substance Matched

#### Matched Pair Analysis (MPA)

**Figure 4:**
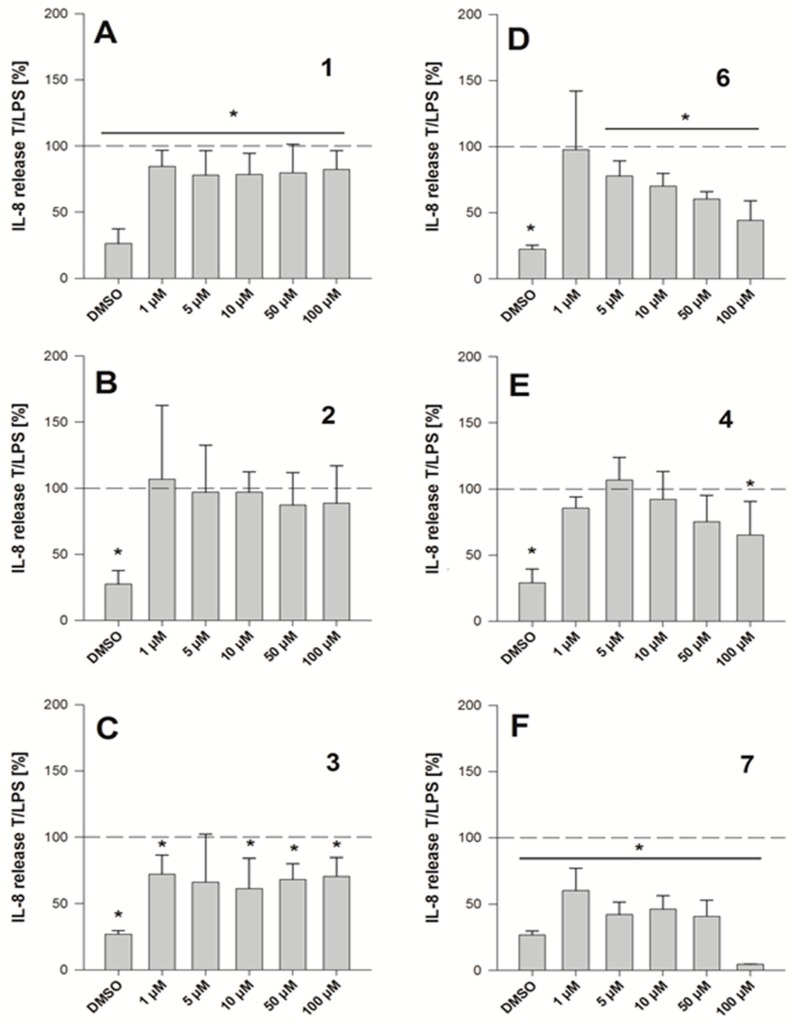
IL-8 release by HGF-1 cells expressed as test over *pg*LPS control (%) for compounds **1**–**4**, **6**, and **7** (**A**–**F**) upon 6 h of co-incubation; data are depicted as average ± SD, *n* = 3–4, technical replicates = 3, *significant differences to *pg*LPS control (*p* < 0.05, Student’s *t*-test)**.**

Figure S1 shows the induction of IL-8 release by *pg*LPS compared to the control as well as the anti-inflammatory effect of 1 µM dexamethasone as a positive control. To compare the inhibitory effects of the compounds, all data are presented in comparison to the *pg*LPS treatment (T/LPS, %). As the groups depicted in Figure 2 showed diverse anti-inflammatory activities, we concentrated on the group closest related to 1 (**2**–**4**, **6**, **7**), subjecting single substances to a matched pair analysis (MPA), with only one structural difference between two compounds, by comparing mean AUCs (Table 1). Figure 4 shows the IL-8 release data for substances **1**–**4**, **6**, and **7** in direct comparison. While compounds **1**, **3**, and **7** already exhibit an anti-inflammatory effect at the lowest tested concentration, compounds **4** and **6** show a concentration dependent inhibition of *pg*LPS-induced IL-8 release. Furthermore, the inhibition of *pg*LPS-induced IL-8 release by 5 µM compound **7** to 42.1 ± 9.58% is comparable to the effect of the positive control, 1 µM dexamethasone, reducing IL-8 release in HGF-1 cells to 37.9 ± 10.1% (Figure S1). In addition to IL-8 release, qPCR experiments for *IL-8* mRNA expression were performed for the selected substances (**2**–**4**, **6**) at 1 µM, 10 µM, and 100 µM after 3 h of incubation to compare to release data (see supplement Figure S4), confirming the concentration dependent activity of compound **6**. However, comparing the individual AUC values of all related compounds, 2′,4,4′,6′-tetrahydroxy 3-methoxydihydrochalcone (**7**) possessed the greatest anti-inflammatory activity (AUC = 3303 ± 816).

For individual structure comparison, we first analyzed the influence of glycosylation (Figure 4A–C) compared to aglyca hesperitin dihydrochalcon (**6**) and hesperitin (**4**) (Figure 4D,E), with the comparison of **1** and **6**, **2** and **4**, and **3** and **4**. In the first two pairwise comparisons, glycosylation did have a more deleterious effect on activity in the A,B-linker system (ΔAUC_1/6_ = 1985), as compared to the bicyclic core structure (ΔAUC_2/4_ = 1371). The attachment of a different disaccharide was tolerated better, diminishing the here tested bioactivity by ΔAUC_3/4_ = 1092. In direct comparison of the glycosylated compounds, we could confirm that hesperidin (**3**, AUC = 6648, Figure 4C) possessed a higher anti-inflammatory activity than neohesperidin (**2**, AUC = 9111, Figure 4B), with a ΔAUC_2/3_ of 2463. The structural difference between these two compounds resides in the disaccharide moiety. Therefore, we hypothesize that the sugar moiety also affects the anti-inflammatory impact of the compounds on *pg*LPS-induced IL-8 release by HGF-1 cells. We could not find a correlation between the effect on *pg*LPS-induced IL-8 release with molecular weight, polarity, surface polarity, or hydrogen bond acceptors/donors. As we were able to test neohesperidin (**2**) in comparison to neohesperidin dihydrochalcone (**1**), a missing link in this group would be the anti-inflammatory evaluation of a hesperidin dihydrochalcone in comparison to hesperidin (**3**), but we were not able to obtain that substance in the present study. Cleavage of the C-ring of hesperitin (**4**) to the dihydrochalcone (A-ring-linker-B-ring)-system (**6**, Figure 4D), instead of the A,C-bicyclic system with a solitary B-ring (Figure 4E), revealed a notable difference in the effect size of ΔAUC_4/6_ = 1795 and hesperitin dihydrochalcon as a very potent compound. Furthermore, changing the substitution pattern at the B-ring from an isovanilloylic to a vanilloylic ring resulted in a ΔAUC_6/7_ = 2642. Thus, 2′,4,4′,6′-tetrahydroxy 3-methoxydihydrochalcone (**7**), the non-glycosylated dihydrochalcon with a vanilloylic B-ring remained the most potent anti-inflammatory substance tested (Figure 4F).

## 3. Discussion

Anti-inflammatory and antioxidant potential of polyphenols are often presented as collateral bioacitivies [5]. Here, our data suggest the more flexible A,B-linker system to be more beneficial for an anti-inflammatory activity than the bicyclic systems. On the other hand, in a QSAR analysis on antioxidant activities by Khlebnikov et al. [29], the more flexible A,B-linker systems were not superior to the bicyclic systems. A higher π-electron delocalization in the bicyclic system would of course be beneficial for radical scavenging effects. Small changes in the molecule structure might have an impact on pharmacodynamics and metabolic transformations which, in turn, might affect the antioxidant and anti-inflammatory activity. Such structure-specific activities of polyphenolic compounds regarding redox properties and anti-inflammatory activities have already been observed in our group for physiological metabolites of substance **18**, namely resveratrol [7,16,30]. However, future studies are needed to elucidate this hypothesis. Another possibility would be a different mode of action of bicyclic and A,B-linker systems due to increased molecular flexibility.

After our initial analysis of the A-ring substituents as shown in Figure 2, substances **7** (trihydroxylated), **10** (dihydroxylated), and **12** (monohydroxylated) were also subjected to individual matched pair analyses (MPA) to evaluate the impact of single changes in molecules that are otherwise identical. ΔAUC_7/10_ (tri- vs dihydroxylated) and ΔAUC_10/12_ (di- vs monohydroxylated) were 1749 and 1888, respectively, making the trihydroxylated A-ring the most potent substitution pattern, with a ΔAUC_7/12_ of 3637. The difference in bioactivity between tri- and di-hydroxylated compounds could also be established for bicyclic compounds **15** and **16**, resulting in ΔAUC_15/16_ of 1722.

To obtain a detailed insight into the impact of B-ring substitution (vanilloyl/isovanilloyl/p-OH-benzyl) on *pg*LPS-stimulated IL-8 release in HGF-1 cells, two direct comparisons were performed. Experiments with compounds **10** and **11** (both with dihydroxylated A-ring) revealed a lower ΔAUC_11/10_ of 574 as opposed to compounds **6** and **7** (ΔAUC_6/7_ = 2642), both with trihydroxylated A-ring, indicating not only that the vanilloyl group was important for the bioactivity in both comparisons, but also that a combination of trihydroxylation of the A-ring with a vanilloyl group as the B-ring resulted in the highest bioactivity in the used in vitro system. As a last step, we increased the electronegativity of the A,B-ring linker by exchanging the keto group for an amide functional moiety. The result was a marked increase in effect size with a ΔAUC_13/12_ of 2577, favoring the amidic function. Ideally, this hypothesis would have been proven by a comparison of **14** with the respective compound, which we unfortunately could not include in this study. The addition of a second hydroxyl group to the A-ring, which according to our earlier MPA analysis should have had positive effects, did on the contrary vastly diminish the effect (ΔAUC_13/14_ = 3195). Here, we hypothesize that for **14** the increased rigidity has a greater impact on the anti-inflammatory effect than the introduction of an additional hydroxyl group. This would be in accordance with the differences in activity we found for the much more rigid bicyclic structures (**2**–**5**, **15**, **16**) compared to compounds with two freely rotating aromatic rings (**1**, **6**–**13**) with ΔAUC_(median, bicyclic)/(median, A,B-linker)_ = 1955.

Compound **18**, representing the compound class of stilbenes, with a well-documented anti-inflammatory activity [16], exhibited a much higher AUC_median_ (6362) compared to the majority of dihydrochalcones tested. Moreover, the shortening of the A,B-linker by one carbon atom was detrimental on the tested bioactivity in this study (ΔAUC_10/17_ = 1326), whereas the removal of the keto function in bicyclic compounds was of little difference with a ΔAUC_4/15_ of 400. For compound **18** (resveratrol), direct molecular interactions have been shown with cyclooxygenases, leukotriene hydrolases, and the transcription factor peroxysome proliferator activating receptor gamma (PPARγ), all of which are involved in inflammation [31]. For the here tested dihydrochalcones, as far as we are aware, no such studies exist yet.

The chosen compounds share close structural similarities but show differences in their anti-inflammatory properties, which might be explained by different molecular mechanisms of action. For selected naturally occurring dihydrochalcones, a direct molecular interaction with cathepsins, enzymes involved in inflammation and metabolic disorders [32], has already been established by Burger et al. [33]. Future studies could, therefore, focus on this direct interaction. Another possibility would be the interaction with transcription factor(s), or a direct pathway inhibition at the TLR, for which dihydro-pyrrolo[2,3-d]pyrimidines have been recently demonstrated to be effective [34]. Concerning a possible interaction with transcription factor Nf-κB, it would be interesting to use the results presented here as a test set for already existing in silico screening systems for small molecule immune-modulators, as published by Tsai and colleagues for NF-κB [35]. Here, the authors focused on the *pg*LPS-induced release of IL-8 as a key cytokine of the immune response in HGF-1 cells. However, future studies may include the effect of the tested dihydrochalcones on the LPS-induced release of other cytokines such as IL-6 or MCP-1, which have been shown to be affected by various natural compounds [6,17]. Our results might also be useful in advancing efforts in the de novo design of anti-inflammatory drugs. It is possible that the anti-inflammatory activity of compounds with a chalcone or dihydrochalcone core, like the CXCL12 neutraligand, as published by Gasparik et al. [36], could also be enhanced by the introduction of the structural features identified in this study.

## 4. Materials and Methods

### 4.1. Chemicals

Fetal bovine serum (FBS) was obtained from Invitrogen (Karlsruhe, Germany), *pg*LPS from InvivoGen (San Diego, CA, USA), the peqGOLD total RNA isolation kit and qPCR plates were purchased from Peqlab (Erlangen, Germany), and the high capacity cDNA reverse transcription kit and fast SYBR green mastermix for PCR experiments were from LifeTechnologies (Carlsbad, CA, USA). ELISA kits for IL-8 were obtained from Merck (Vienna, Austria). All other chemicals were from Sigma-Aldrich (Vienna, Austria).

### 4.2. Compounds

**Figure A1:**
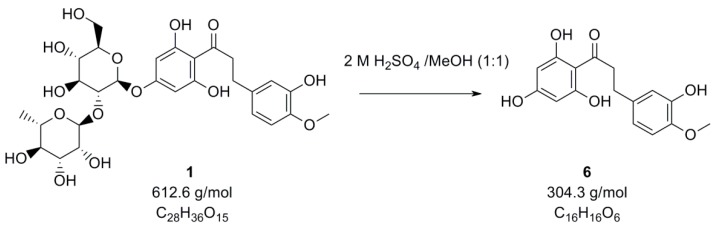
Preparation of compound **6**.

**Figure A2:**
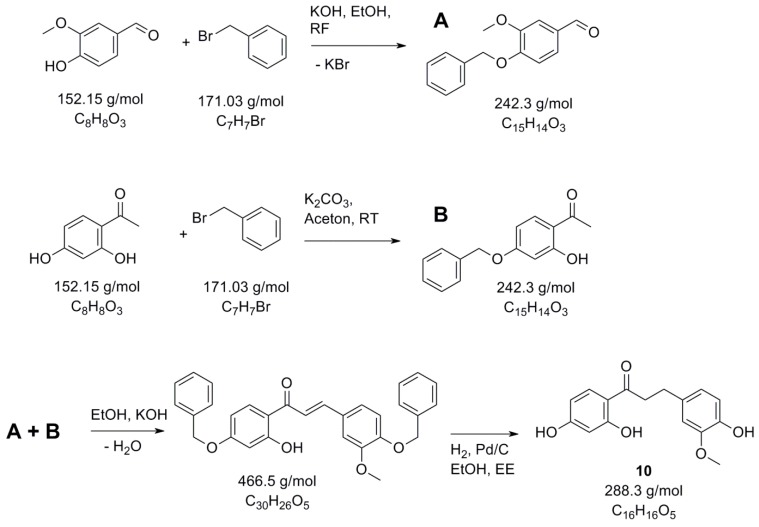
Synthesis of compounds **A**, **B**, and **10**.

**Figure A3:**
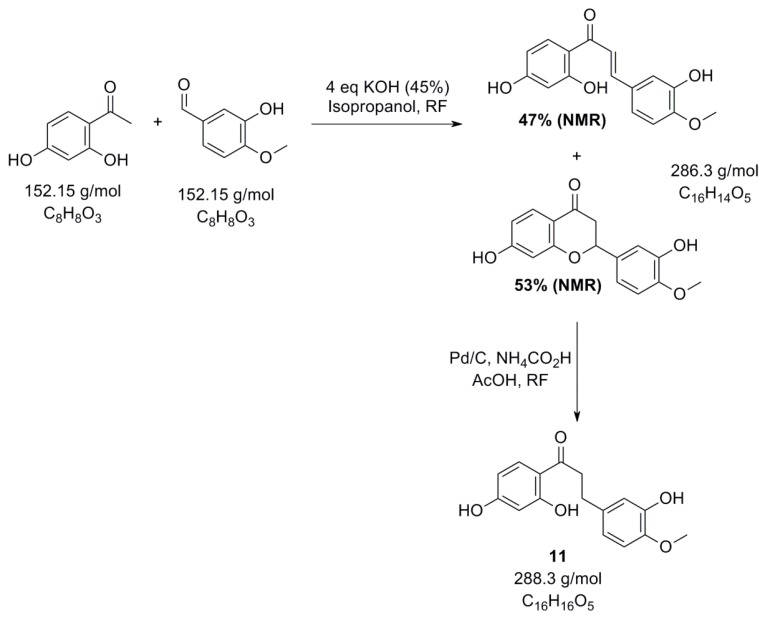
Synthesis of compound **11**.

**Figure A4:**
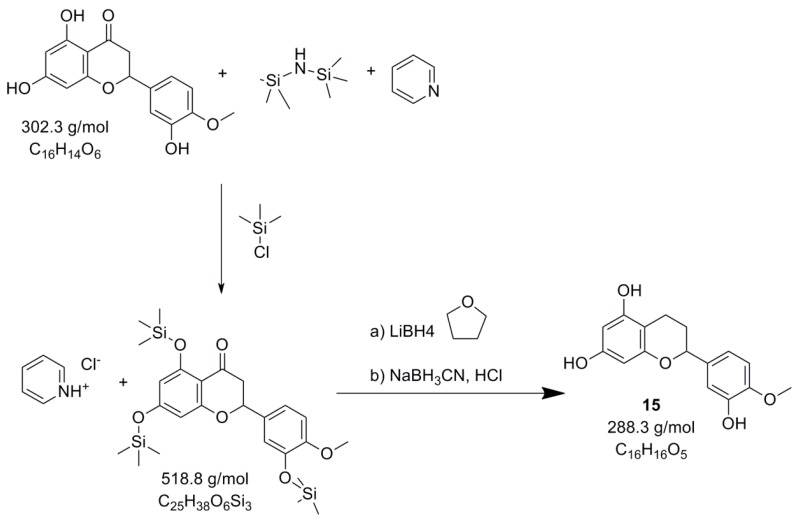
Preparation of compound **15**.

**Figure A5:**
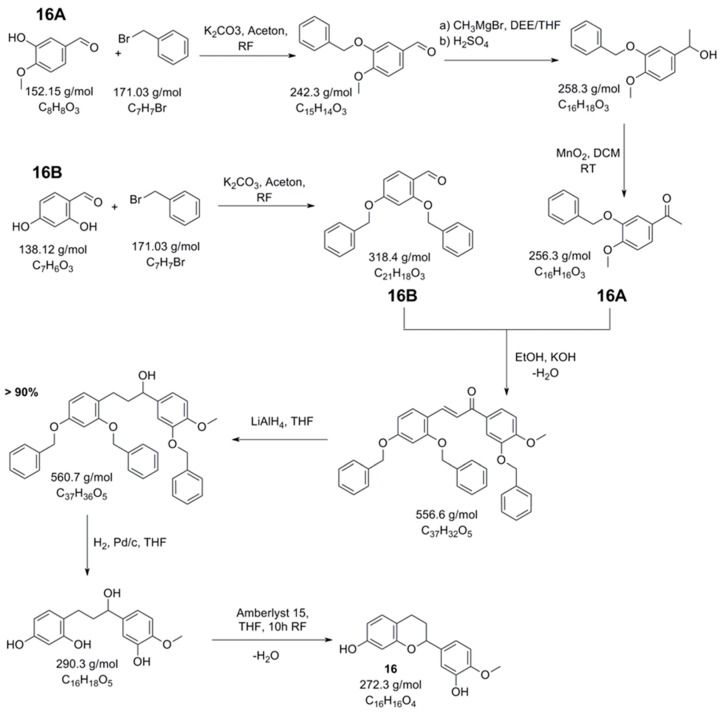
Synthesis of compounds **16A**, **16B**, and **16**.

Compounds **1**–**5**, **8**, and **18** were obtained from Sigma-Aldrich (Munich, Germany). Compounds **13** and **14** were synthesized according to Ley et al. [37]. Compounds **7**, **9**, **12**, and **17** were synthesized following the protocol published by Ley et al. [38]. Synthesis of compounds **6** (Figure A1), **10** (Figure A2), **11** (Figure A3), **15** (Figure A4), and **16** (Figure A5), as well as NMR data and purities of all synthesized compounds, are presented in Appendix A. Synthetic dihydrochalcones were tested alongside well-characterized natural compounds (**2**–**5**, **8**, **9**, and **18**) [13,35,39,40,41] for a more comprehensive evaluation of the anti-inflammatory effect.

### 4.3. Cell Culture

The Human Gingival Fibroblasts (HGF-1) cell line was obtained from the American Type Culture Collection at passage 15. Cultures were split twice a week and for experiments, cells were only used up to passage 24. HGF-1 cells were cultivated in Dulbecco’s Modified Eagle Medium (DMEM), supplemented with 10% fetal bovine serum, 100 U/mL, penicillin, 0.1 mg/mL streptomycin, and 8 mM glutamine in a humidified incubator with 5% CO_2_ at 37 °C. The specified medium was used in all experiments. For incubations, cells were seeded at a density of 5000 cells per well (96 well plates) in 200 µL medium and allowed to attach and grow for three days.

Cells were then incubated in triplicates with *Porphyromonas gingivalis* LPS (10 µg/mL, *pg*LPS) alone (*pg*LPS control) or co-incubated with five different concentrations of a test compound (1–100 µM, 100 µL incubation medium) for 6 h. Compounds were dissolved in DMSO to a concentration of 100 mM. Dilutions were then prepared for additional concentrations of 50 mM, 10 mM, 5 mM, and 1 mM in 100% DMSO. Stocks were always prepared freshly on the day of incubation. The final solvent concentration was 0.1% DMSO in all experiments. The cell supernatant was then used to determine the IL-8 release (see Section 4.5) and the cells were tested for their viability upon incubation (see Section 4.4).

### 4.4. Cell Vitality

Cell vitality was measured in 96 well plates after 6 h incubation using the MTT assay. After removal of cell supernatant, cells were incubated with 100 µL of MTT reagent (5 mg/mL in PBS, 1:6 dilution with serum-free medium) for 30–45 min. After removing the excessive MTT reagent, the blue formazan crystals formed within the viable cells were dissolved in 150 µL DMSO, and absorption was measured at 570 nm, with reference wavelength 650 nm with an Infinite M200 plate reader (Tecan, Austria). Viability was calculated as compared to cells incubated with a solvent-free medium.

### 4.5. Measurement of IL-8 in Cell Culture Supernatants

IL-8 levels in cell culture supernatants collected upon treatment (see Section 4.3) were measured by ELISA according to the manufacturer’s protocol (Merck Millipore, Darmstadt, Germany) using an Infinite M200 plate reader (Tecan, Austria).

### 4.6. Analysis of IL-8 mRNA Expression in pgLPS-Stimulated HGF-1 Cells Incubated with Selected Dihydrochalcones

For the detection of mRNA analysis, the 3 h *pg*LPS-stimulated and incubated HGF-1 cells were lyzed and mRNA was isolated using a commercial kit (Peqlab). Transcribed cDNA was used to perform the qPCR analysis with PPIA and GAPDH as reference genes (StepOnePlus, Applied Biosystems) as previously described [6]. Based on the C_T_-values, the starting material N_0_ was calculated by LinRegPCR. N_0_-values of IL-8 were normalized to the N_0_-values of the reference genes. The normalized N_0_-levels upon co-incubation with *pg*LPS and the compound of interest were related to the normalized N_0_-levels of *pg*LPS-stimulated cells (100%).

### 4.7. Statistical Analysis

Data represent averages ±SD and were analyzed using SigmaPlot version 13.0 (SystatSoftware, Erkrath, Germany). Prior to comparative testing, all sets were normality tested (Shapiro–Wilk). If normality was passed, a Student’s *t*-test was performed (*, *p* < 0.05) against *pg*LPS control; if equal variance testing failed, a Wilcoxon–Mann–Whitney Rank Sum test was performed.

## 5. Conclusions

In conclusion, we determined for the here tested dihydrochalcone structure fragments, the most favorable combination is a trihydroxylated A-ring moiety with a vanilloylic B-ring moiety regarding a *pg*LPS-induced IL-8 release reducing effect in human gingival fibroblasts (HGF-1 cells), as a well-established model of gingivitis. Additionally, the introduction of an electronegative atom in the A,B-linker chain may improve the reduction of a *pg*LPS-induced IL-8 release as shown by the comparison of compounds **12** and **13**. We, therefore, propose substances **7** and **13** to be used as lead structures in future assays on the anti-inflammatory activity of dihydrochalcones in oral inflammation.

## Figures and Tables

**Table 1:** Basic core structures with labels, numbering, common names (if applicable), structures, and calculated areas under the curve (AUCs) as the sum of trapezoid areas under the dose–response curve of interleukin-8 (IL-8) values (normalized to *Porphyromonas gingivalis* lipopolysaccharide (*pg*LPS) control) from 1 µM to 100 µM for tested substances **1**–**18**.

No	Common Name	Structure	AUC (1–100 µM) Mean ±SD
	A,C-bicyclic system with solitary Bring	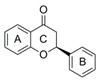	-
-	A-ring-linker-B-ring system	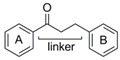	-
1	Neohesperidin dihydrochalcone	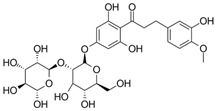	7930 ± 1730
2	Neohesperidin	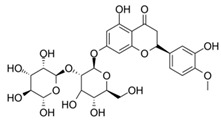	9111 ± 2497
3	Hesperidin	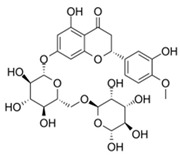	6648 ± 1589
4	Hesperetin	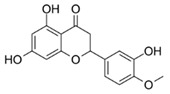	7740 ± 1782
5	Liquiritigenin	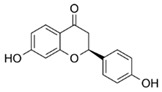	8942 ± 2450
6	Hesperetin dihydrochalcone	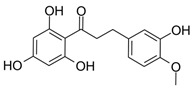	5945 ± 969
7	2′,4,4′,6′-tetrahydroxy 3-methoxydihydrochalcone	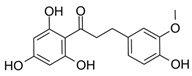	3303 ± 816
8	Phloretin	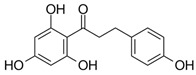	7872 ± 629
9	Davidigenin	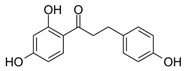	6527 ± 1625
10	2′,4,4′-trihydroxy 3-methoxydihydrochalcone	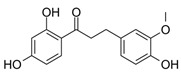	5052 ± 596
11	2′,3,4′-trihydroxy 4-methoxydihydrochalcone	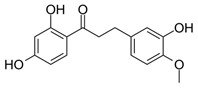	5626 ± 614
12	4,4′-dihydroxy 3-methoxydihydrochalcone-	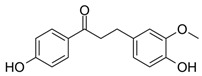	6940 ± 950
13	4-hydroxybenzoic acid *N*-vanillylamide	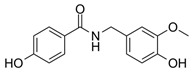	4363 ± 696
14	2,4-dihydroxybenzoic acid *N*-vanillylamide	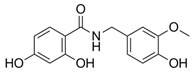	7558 ± 973
15	3′,5,7,-trihydroxy 4′-methoxyflavan	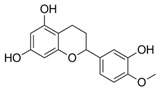	7341 ± 942
16	3′,7-dihydroxy 4′-methoxyflavan	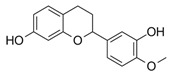	9063 ± 1511
17	3′methoxy 2,4,4′-trihydroxydeoxybenzoin	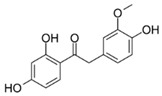	6378 ± 1389
18	Resveratrol	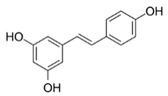	6512 ± 1761

## Supplementary Materials

The analysis of IL-8 release and *IL-8* mRNA expression are available online. Figure S1: Results for incubations with compounds **1**–**6** at 1 µM, 5 µM, 10 µM, 50 µM, and 100 µM in co-incubation with *pg*LPS (10 µg/mL) in HGF-1 cells after 6 h, Figure S2: Results for incubations with compounds **7**–**12** at 1 µM, 5 µM, 10 µM, 50 µM, and 100 µM in co-incubation with *pg*LPS (10 µg/mL) in HGF-1 cells after 6 h, Figure S3: Results for incubations with compounds **13**–**18** at 1 µM, 5 µM, 10 µM, 50 µM, and 100 µM in co-incubation with *pg*LPS (10 µg/mL) in HGF-1 cells after 6 h, Figure S4: Results for mRNA expression upon incubation with compounds **2**–**4** and **6** at 1 µM, 10 µM, and 100 µM in co-incubation with *pg*LPS (10 µg/mL) in HGF-1 cells after 3 h.

## Appendix A. Preparation and Structure Elucidation of Compounds 6, 10, 11, 15, and 16

### Appendix A.1. Compound ***6***

Compound **6** was prepared by dissolving compound **1** (Neohesperidin dihydrochalcone) in MeOH/2M H_2_SO_4_ (1:1, *v*/*v*) and refluxing the mixture for 6 h (Figure A1). The reaction mixture was neutralized with 2M NaOH for precipitation. After filtration, the precipitate was washed with water and dried at 40 °C under high vacuum. Compound **6** was obtained as a colorless solid with a yield of 77%.

Identity and purity was assessed by NMR: ^1^H-NMR (400 MHz, DMSO-d6): δ 12.25 (s, 2H), 10.36 (s, 1H), 8.79 (s, 1H), 6.80 (d, *J* = 8.2 Hz, 1H), 6.66 (d, *J* = 2.1 Hz, 1H), 6.59 (dd, *J* = 8.2, 2.2 Hz, 1H), 5.81 (s, 2H), 3.72 (s, 3H), 3.22 (dd, *J* = 8.5, 6.9 Hz, 2H), 2.74 (dd, *J* = 8.5, 6.9 Hz, 2H). ^13^C NMR (100 MHz, DMSO-*d*6): δ 203.97, 164.51, 164.12 (2C), 146.19, 145.70, 134.13, 118.59, 115.61, 112.24, 103.60, 94.54 (2C), 55.59, 45.11, 29.42; melting point (mp, uncorrected): 144–147 °C under decomposition.

### Appendix A.2. Compound ***10***

Compound **10** was synthesized using compounds **A** and **B**. The synthesis of compounds **A** and **B** and compound **10** are shown in Figure A2.

Synthesis of compound **A**: One equivalent (eq) 4-hydroxy-3-methoxy-benzaldehyde and 2.2 eq benzylbromide were dissolved in ethanol containing 2 eq potassium hydroxide and refluxed (RF) for 8 h. After cooling the mixture to room temperature and evaporating to dryness, the residue was dissolved in ethyl acetate and washed three times with H_2_O. The organic phase was then dried with anhydrous sodium sulfate and evaporated to dryness. The crude product (74.9% 4-benzyloxy-3-methoxybenzaldehyde as determined by GC) was recrystallized from ethanol, yielding 81.4% of 4-benzyloxy-3-methoxybenzaldehyde with a purity of 100% (GC).

Synthesis of compound **B**: One eq 2′,4′-dihydroxyacetophenone was dissolved in acetone and 2.2 eq of potassium carbonate were added under stirring, followed by the addition of 1.1 eq benzyl bromide. The mixture was stirred for 20 h at room temperature, filtered using a Büchner funnel, and the separated salt residues were washed with acetone. The collected filtrate was then evaporated to dryness and taken up in ethyl acetate. The organic phase was washed with a sodium chloride solution (10%, *w*/*v*), dried with anhydrous sodium sulfate, and evaporated to dryness. The resulting crude crystalline product was dissolved in hexane/acetone (3:1, *v*/*v*) under reflux. After cooling down, the resulting precipitate was isolated via filtration and after drying identified as 2′-hydroxy-4′-benzyloxy-acetophenone (99.9% purity as determined by GC, yield 70.7%).

Synthesis of compound **10**: One eq A and one eq B were dissolved in ethanol. A total of 1.2 eq potassium hydroxide solution (15%, *w*/*v*) were added dropwise under constant cooling over 5 min. After stirring at 25 °C overnight, the mixture was cooled down to 0–5 °C and stirred for another 1.5 h. The mixture was then acidified with 2 eq glacial acetic acid and precipitated with H_2_O by stirring at 0–5 °C for 1 h. The precipitate was filtered, washed 3 times with ethanol (50%), and dried at 80 °C. The dried precipitate was then dissolved in a mixture (1:1) of tetrahydrofuran and ethanolic potassium hydroxid solution (10%, *w*/*v*) and refluxed for 18 h. After cooling, the mixture was again acidified with glacial acetic acid and precipitated with H_2_O by stirring at 0–5 °C for 1 h. The crude precipitate was then recrystallized from acetone. The refined product was then dissolved in ethyl acetate/ethanol (2:1, *v*/*v*), hydrogenated using Pd/C 5% (for approximately 6 h). After the removal of the catalyst via filtration over SiO_2_, the solvent was evaporated, and the crude product was recrystallized from hexane/ethyl acetate.

The synthesis of compound **10** resulted in a yield of 45.4%, with a purity of 97.7% as determined by NMR. ^1^H-NMR (400 MHz, DMSO-*d*6): δ ppm 12.65 (s, OH), 7.81 (d, *J* = 8.9 Hz, 1H), 6.83 (d, *J* = 1.5 Hz, 1H), 6.66 (d, *J* = 7.9 Hz, 1H), 6.63 (dd, *J* = 8.0, 1.7 Hz, 1H), 6.36 (dd, *J* = 8.8, 2.3 Hz, 1H), 6.25 (d, *J* = 2.3 Hz, 1H), 3.74 (s, 3H), 3.65–3.46 (m, OH), 3,23 (t, *J* = 7.6 Hz, 2H), 2.83 (t, *J* = 7.6 Hz, 2H), 2.50 (s, OH), 1.26 (dd, *J* = 18.3, 4.0 Hz, OH), 0.9–0.82 (m, OH). ^13^C NMR (101 MHz, DMSO) δ ppm 203.83, 164.67, 164.15, 147.28, 144.56, 132.95, 131.60, 120.32, 115.15, 112.55, 112.44, 108.05, 102.29, 55.44, 40.04, 39.83, 39.62, 39.41, 39.20, 38.99, 38.79, 30.86, 29.49, 21.97, 13.87; mp (uncorrected): 147–149 °C.

### Appendix A.3. Compound ***11***

One eq isovanillin and one eq 2′,4′-dihydroxyacetophenone were dissolved in isopropanol and 4 eq potassium hydroxide solution (45%, *w*/*v*) were added continuously over 10 min (Figure A3). The reaction mixture was heated up to 80 °C under stirring and refluxed for 8 h. Half of the reaction volume was removed using a Claisen condenser and the remaining mixture was diluted with 4 volumes of H_2_O and acidified with 6 eq glacial acetic acid under stirring for precipitation. The product mixture (286.3 g/mol, 47%/53% as determined by NMR) was filtered, washed with H_2_O, and dried at 70 °C. The obtained product mixture was taken up in glacial acetic acid and reacted with 2 eq ammonium formiate and Pd/C 5% under reflux conditions for 7 h. After separation of the catalyst via filtration, the glacial acetic acid was evaporated, the residue was dissolved with ethyl acetate, washed twice with water, dried over Na_2_SO_4_, and the solvent was removed under vacuum. The crude product was then dissolved in isopropanol/H_2_O (3:7, *v*/*v*) and stirred overnight at room temperature.

The formed crystals were washed 3 times with H_2_O and dried at 70 °C, resulting in compound **11** as a pearl-white solid (purity of 99.3%, as determined by NMR). ^1^H-NMR (400 MHz, DMSO-*d_6_*): δ ppm 12.62 (s, OH), 7.79 (d, *J* = 8.9 Hz, 1H), 6.80 (d, *J* = 8.2 Hz, 1H), 6.68 (d, *J* = 2.1 Hz, 1H), 6.62 (dd, *J* = 8.2, 2.1 Hz, 1H), 6.36 (dd, *J* = 8.8, 2.4 Hz, 1H), 6.25 (d, *J* = 2.3 Hz, 1H), 3.72 (s, 3H), 3,20 (t, *J* = 7.5 Hz, 2H), 2.79 (t, *J* = 7.5 Hz, 2H). ^13^C NMR (101 MHz, DMSO) δ ppm 203.61, 164.67, 164.12, 146.19, 145.80, 133.50, 132.87, 118.64, 115.69, 112.42, 112.20, 108.06, 102.30, 55.60, 40.03, 39.82, 39.61, 39.41, 39.20, 39.05, 38.99, 38.78, 29.04; mp (uncorrected): 118 °C under decomposition.

### Appendix A.4. Compound ***15***

According to a known procedure [42], 1.0 eq hesperetin was taken up in 12.4 eq pyridine, and 4.4 eq of bis(trimethylsilyl)amine were added (Figure A4). Subsequently, 4.7 eq of trimethylsilyl chloride were added dropwise, and the mixture was stirred for another half hour. Pyridin was evaporated and 94 eq toluol were added to the remaining reaction volume. The mixture was filtered and evaporated. The remaining oily residue was taken up in 31 eq tetrahydrofuran. Subsequently, 0.5 eq lithium borohydride was carefully added, and after stirring for 1 h, 1 eq sodium borohydride cyanide was added, as well as 0.5 mg methyl orange as an indicator. Hydrochloric acid (1 M) was added dropwise until a stable color change was detected. THF was evaporated and the remaining mixture was extracted three times with ethyl acetate. The organic phase was then washed with hydrochloric acid (1 M), H_2_O, and saturated sodium chloride solution, and evaporated to dryness.

The product was purified (>99% as determined by NMR) by column chromatography with a solvent mixture of ethyl acetate/pentane (1:1). ^1^H-NMR (400 MHz, DMSO-*d_6_*): δ ppm 9.16 (s, OH), 8.97 (s, 1H), 8.92 (s, 1H), 6.89 (d, *J* = 8.3 Hz, 1H), 6.81 (d, *J* = 1.9 Hz, 1H), 6.76 (dd, *J* = 8.3, 1.9 Hz, 1H), 5.89 (d, *J* = 2.3 Hz, 1H), 5.70 (d, *J* = 2.3 Hz, 1H), 4.81 (dd, *J* = 9.7, 1.6 Hz, 1H), 3.75 (s, 3H), 2.49 (dd, *J* = 9.4, 4.7 Hz, 2H), 2.06–1.96 (m, 1H), 1.89–1.74 (m, 1H). ^13^C NMR (101 MHz, DMSO) δ ppm 156.19, 156.02, 155.94, 146.97, 146.23, 134.36, 116.75, 113.34, 111.90, 100.12, 94.86, 94.16, 76.19, 55.55, 40.02, 39.81, 39.60, 39.39, 39.18, 38.97, 38.77, 28.93, 18.77; mp (uncorrected): 164–166 °C.

### Appendix A.5. Compound ***16***

To synthesize compound **16**, compounds **16A** and **16B** needed to be synthesized first (Figure A5).

Synthesis of compound **16A**: One eq 3-hydroxy-4-methoxybenzaldehyde was dissolved in acetone and 1.2 eq potassium carbonate were added under reflux, followed by the dropwise addition of 1 eq benzyl bromide over 30 min. The mixture was refluxed for 14 h, diluted with H_2_O, and extracted with ethyl acetate. The organic phase was then washed with H_2_O and sodium chloride solution, dried with sodium sulfate and evaporated to dryness to yield 3-benzyloxy-4-methoxybenzaldehyde (97.1% purity as determined by GC). The aldehyde reacted with methylmagnesium bromide in diethyl ether/tetrahydrofuran. After quenching the reaction with ice and sulfuric acid (10%, pH < 2) under nitrogen gas, the mixture was diluted with ethyl acetate. The organic phase was washed with H_2_O, sodium carbonate solution, and sodium chloride solution, and evaporated to dryness, yielding 1-(3-benzyloxy-4-methoxyphenyl)ethanol (purity 84.6% as determined by GC). The resulting alcohol was oxidized in dichloromethane using manganese (IV) oxide at room temperature for 144 h. After removing manganese (IV) oxide by filtration, the filtrate was run through a SiO_2_-filled column and evaporated to dryness. The product was recrystallized from hexane/acetone (4:1, *v*/*v*) and yielded 1-(3-benzyloxy-4-methoxyphenyl)ethanone (16A, purity 99.3% as determined by GC). The overall yield was 34.4% over three steps.

Synthesis of compound **16B**: One eq 2′,4′-dihydroxybenzaldehyde was dissolved in acetone and 2.4 eq potassium carbonate were added under reflux, followed by the dropwise addition of 2.1 eq benzyl bromide over 30 min. The mixture was refluxed for 10 h and cooled down to room temperature. After removal of the precipitated salts by filtration, the filtrate was evaporated to dryness. The crude product was then recrystallized from acetone (97.8% purity as determined by GC).

Synthesis of compound **16**: One eq **16A** and one eq **16B** were dissolved in ethanol and 0.5 eq potassium hydroxide was added at room temperature. During refluxing for 12 h, precipitation could already be observed. Ethanol/H_2_O (1:1, *v*/*v*) was added and the mixture was vacuum filtered. The precipitate was washed 4 times with Ethanol/H_2_O (1:1, *v*/*v*) and dried at 70 °C. One eq of precipitate was then dissolved in anhydrous tetrahydrofuran, cooled to 10 °C under inert gas, and 0.5 eq of lithium aluminium hydride dissolved in tetrahydrofuran was added dropwise, while keeping the reaction temperature under 20 °C. After stirring for 3 h at room temperature, the reaction mixture was then again cooled to 0 °C, and the reaction was quenched by adding H_2_O and sodium hydroxide solution (15%, *v*/*w*). After the addition of sodium sulfate, the mixture was stirred for another half hour. The reaction mixture was filtered and the filtrate was evaporated to dryness.

The intermediate compound was hydrogenated in tetrahydrofuran with Pd/C 5% as a catalyst for 12 h and evaporated to dryness after filtration. For cyclization, the resulting product was refluxed for 10 h in tetrahydrofuran with Amberlyst 15, filtered, and evaporated to dryness. After Flash-LC purification (Silica 60, solvent hexane/ethyl acetate 3:2, *v*/*v*) the final product was recrystallized from hexane/acetone (2:1, *v*/*v*), and yielded 33.0% of compound **16** over four steps as an off-white solid (purity >95% as determined by NMR). ^1^H-NMR (400 MHz, DMSO-*d_6_*): δ ppm 9.07 (s, OH), 6.89 (d, *J* = 8.3 Hz, 1H), 6.84 (d, *J* = 8.3 Hz, 1H) 6.82 (d, *J* = 2.1 Hz, 1H), 6.77 (dd, *J* = 8.3, 2.0 Hz, 1H), 6.28 (dd, *J* = 8.2, 2.4 Hz, 1H), 6.19 (d, *J* = 2.4 Hz, 1H), 4.89 (dd, *J* = 9.8, 2.1 Hz, 1H), 3.76 (s, 3H), 2.78 (ddd, *J* = 16.3, 11.0, 5.8 Hz, 1H), 2.57 (dt, *J* = 15.9, 4.4 Hz, 1H), 2.04 (ddd, *J* = 13.5, 5.2, 2.7 Hz, 1H), 1.95–1.80 (m, 1H). ^13^C NMR (101 MHz, DMSO) δ ppm 156.35, 155.26, 147.02, 146.27, 134.22, 129.72, 116.74, 113.32, 112.09, 111.92, 107.83, 102.63, 76.48, 55.56, 40.03, 39.82, 39.61, 39.40, 39.20, 38.99, 38.78, 29.32, 23.59; mp (uncorrected): 163–165 °C.
