# Supplementary material for: Identification of Interleukin-8-Reducing Lead Compounds Based on SAR Studies on Dihydrochalcone-Related Compounds in Human Gingival Fibroblasts (HGF-1 cells) In Vitro
Source: Molecules. 2020 Mar 18;25(6):1382. doi: 10.3390/molecules25061382 (PMC7144391; doi:10.3390/molecules25061382)
Supplement: Supplementary file 1 [file molecules-25-01382-s001.pdf]

molecules

Supplemental material

Identification of Interleukin 8-Reducing Lead Compounds Based on SAR Studies on Dihydrochalcone Related Compounds in Human Gingival Fibroblasts

Katharina Schueller [1], Joachim Hans [2], Stefanie Pfeiffer [1], Jessica Walker [1,3,*], Jakob P. Ley [2] and Veronika Somoza [1,4]

[1] Department of Physiological Chemistry, Faculty of Chemistry, University of Vienna, Althanstraße 14, 1090 Vienna, Austria; Katharina.zakovsek@gmx.at (K.S.); Stefanie.pfeiffer88@gmx.at (S.P.); veronika.somoza@univie.ac.at (V.S.)
[2] Symrise AG, Mühlenfeldstraße, 37603 Holzminden, Germany; joachim.hans@symrise.com (J.H.); jakob.ley@symrise.com (J.P.L.)
[3] Department of Analytical Chemistry, Faculty of Chemistry, University of Vienna, Währinger Straße 38, 1090 Vienna, Austria
[4] Leibniz Institute for Food Systems Biology at the Technical University of Munich, Lise-Meitner-Straße 34, 85354 Freising, Germany
* Correspondence: jessica.walker@univie.ac.at; Tel.: +43-4277-52337; Fax: +43-1-4277-852337

1. Measurement of IL-8 in Cell Culture Supernatant

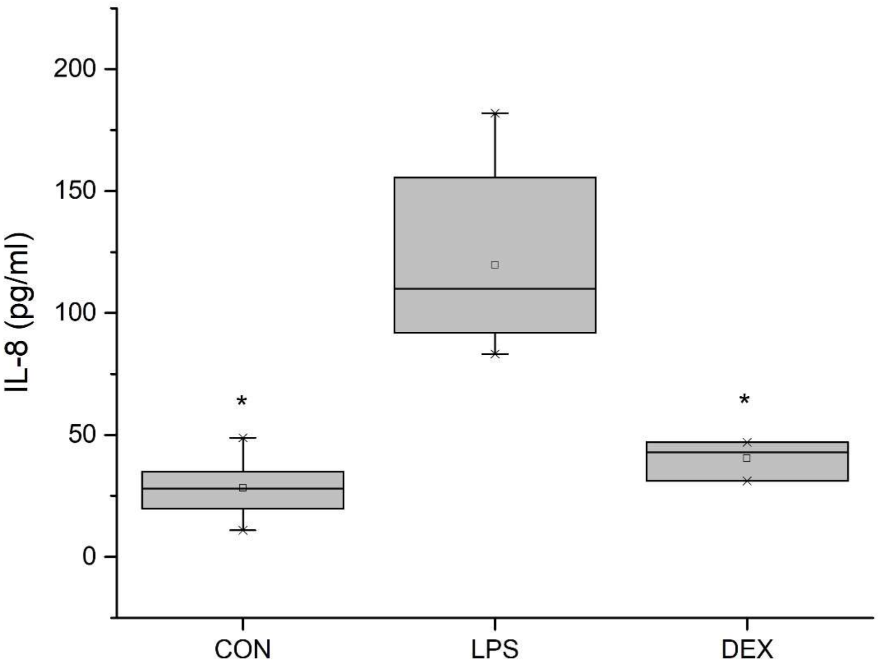

Figure S1. IL-8 release from HGF-1 cells upon 6 h treatment with 0.1% DMSO (CON), 10 ng/ml pgLPS (LPS) and 10 ng/ml pgLPS in combination with 1 μM dexamethasone (DEX) serving as an anti-inflammatory control. Significant differences (p < 0.05) to *pg*LPS control are marked with *, as determined by Student's *t*-test.

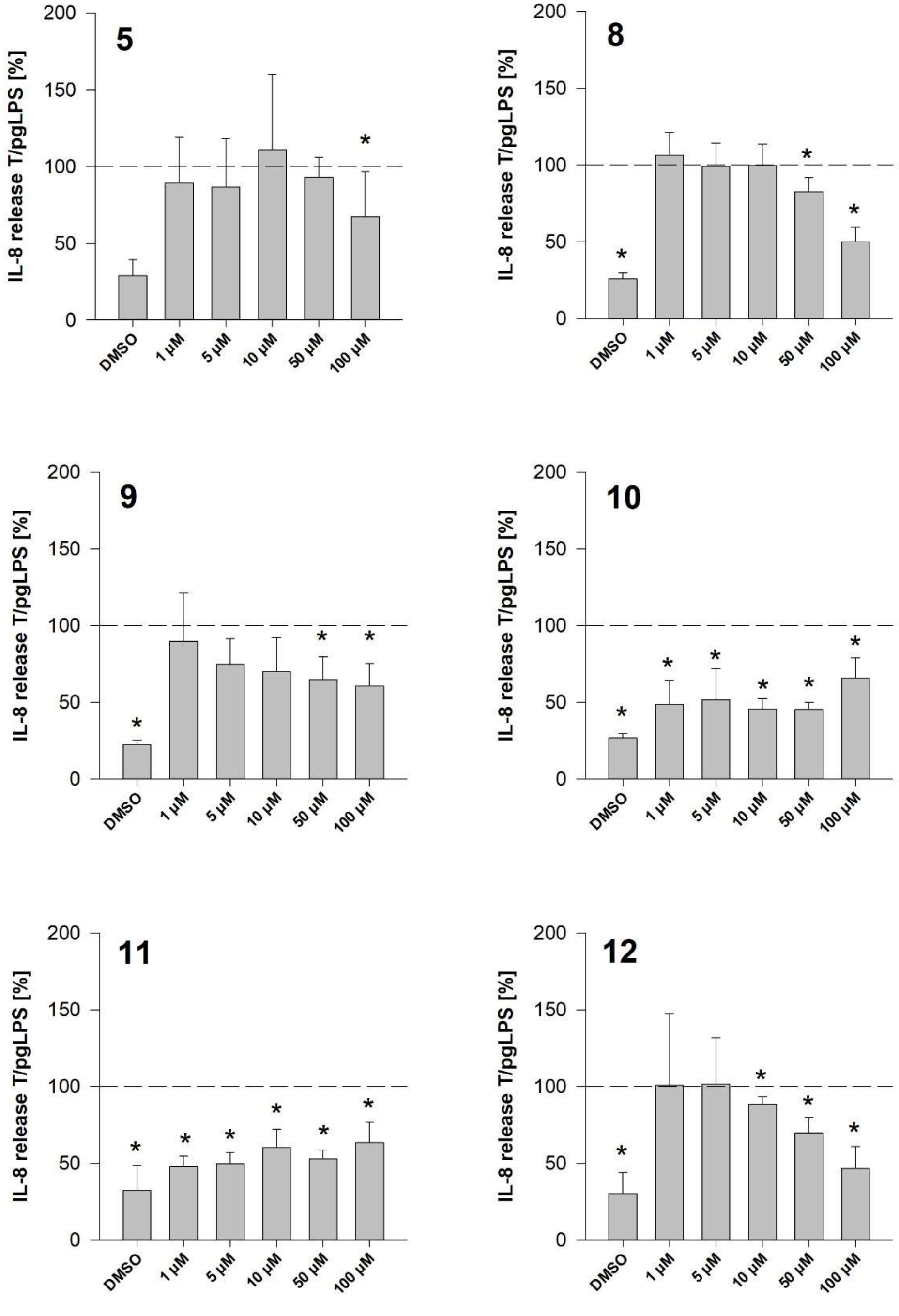

Figure S2. Results for incubations with compounds **5, 8–12** at 1, 5, 10, 50 and 100 µM in co-incubation with *pg*LPS (10 µg/ml) in HGF-1 cells after 6 h. Data are depicted as average ±SD of T/*pg*LPS in %; the dashed line signifies the 100% *pg*LPS control. Significant differences ($p < 0.05$) to *pg*LPS control are marked with *, as determined by Student's *t*-test.

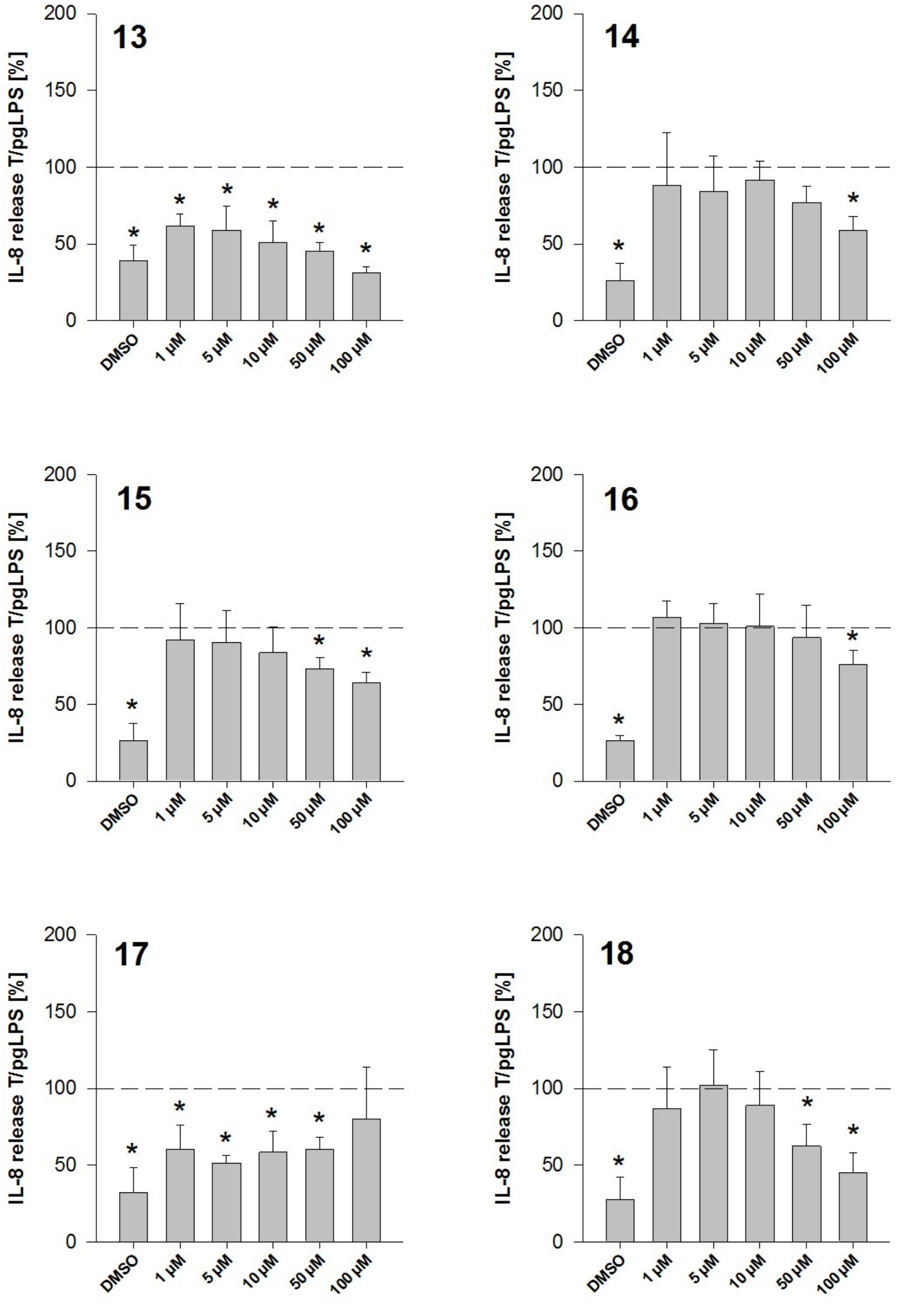

Figure S3. Results for incubations with compounds **13**–**18** at 1, 5, 10, 50 and 100 µM in co-incubation with *pg*LPS (10 µg/ml) in HGF-1 cells after 6 h. Data are depicted as average ± SD of T/*pg*LPS in %; the dashed line signifies the 100% *pg*LPS control. Significant differences ($p < 0.05$) to *pg*LPS control are marked with *, as determined by Student's *t*-test.

2. Analysis of IL-8 (CXCL-8) mRNA Expression in *pg*LPS-Stimulated HGF-1 Cells Incubated with Selected Dihydrochalcones

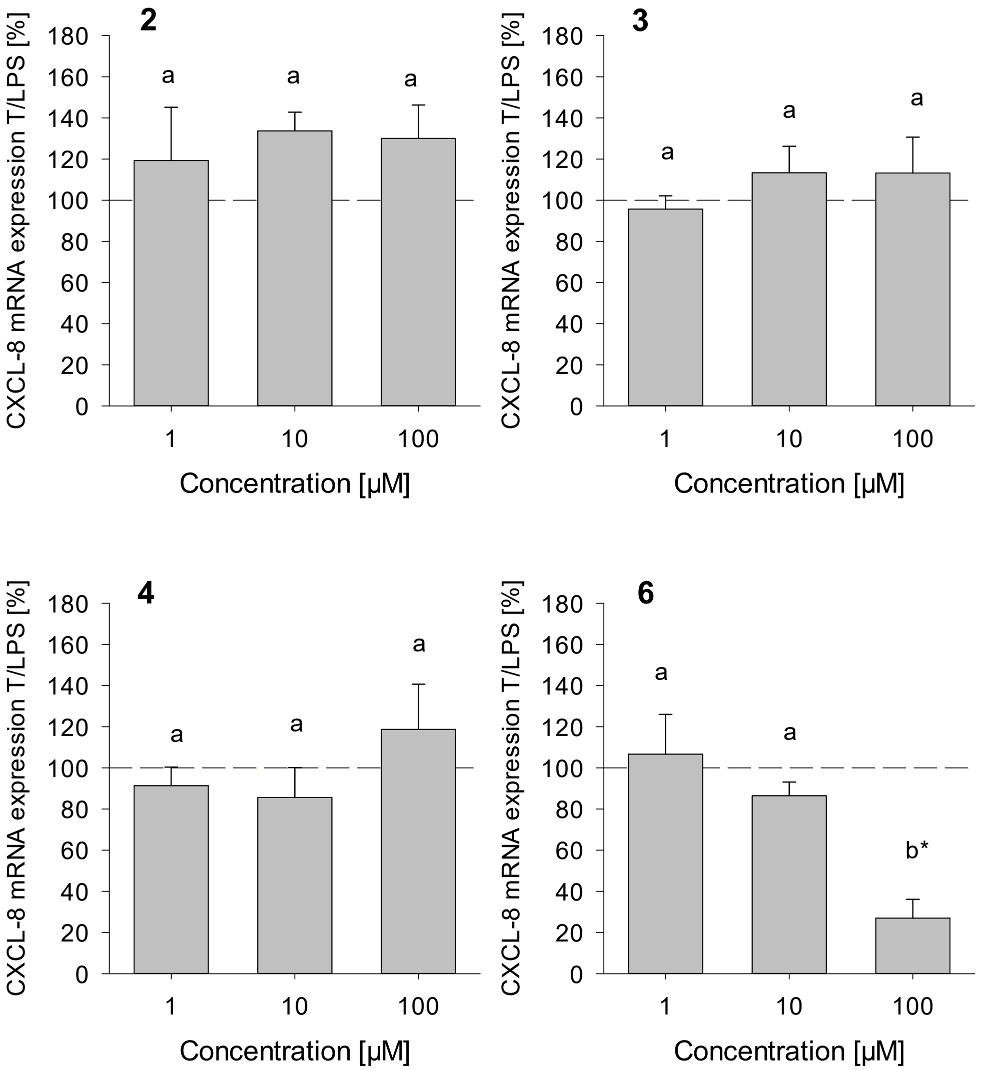

Figure S4. Results for mRNA expression upon incubation with compounds **2**–**4** and **6** at 1, 10 and 100 µM in co-incubation with *pg*LPS (10 µg/ml) in HGF-1 cells after 3 h. Data are depicted as average ± SD of T/*pg*LPS in %; the dashed line signifies the 100% *pg*LPS control. Significant differences ($p < 0.05$) to *pg*LPS control are marked with * and [a,b] among treatment as determined by one-way ANOVA with Tukey post-hoc analysis.
